# Three‐Dimensional Estimation of the Femoral Head Center From Trochanteric Landmarks in Japanese Female Patients With Developmental Dysplasia of the Hip

**DOI:** 10.1111/os.70359

**Published:** 2026-06-22

**Authors:** Norio Imai, Atsushi Sakagami, Daisuke Homma, Yuki Komuta, Yuki Hirano, Yoji Horigome, Hiroyuki Kawashima

**Affiliations:** ^1^ Division of Comprehensive Musculoskeletal Medicine Niigata University Graduate School of Medical and Dental Sciences Niigata Japan; ^2^ Department of Orthopedic Surgery Niigata Bandai Hospital Niigata Japan; ^3^ Division of Orthopedic Surgery, Department of Regenerative and Transplant Medicine Niigata University Graduate School of Medical and Dental Sciences Niigata Japan

**Keywords:** estimation, femoral head center, formula, greater trochanter tip, hip developmental dysplasia, lesser trochanter tip

## Abstract

**Introduction:**

Although pelvic landmarks have traditionally been used to estimate the femoral head center (FC), their reliability may be limited in patients with developmental dysplasia of the hip (DDH). In contrast, femoral‐based reference methods have been insufficiently investigated. This study aimed to evaluate the feasibility and clinical utility of estimating the FC location in DDH using a three‐dimensional model derived from trochanteric landmarks.

**Methods:**

We retrospectively analyzed 128 femurs from 84 female patients with DDH (mean age, 36.9 years) who underwent curved periacetabular osteotomy (CPO) from April 1, 2010, to September 30, 2020, and had no symptoms involving the spine or knee. The FC was estimated using multiple regression models based on the three‐dimensional coordinates (*x*, *y*, and *z*) of the greater and lesser trochanter tips. Differences between the estimated and actual FC positions were assessed along all three axes.

**Results:**

Correlation coefficients between the estimated and actual FC ranged from 0.725 to 0.875 across the three directions. The mean absolute error was 2–3 mm, with greater errors observed in the anteroposterior direction than in the craniocaudal direction. An estimation error within 3 mm may be considered relatively small in the context of clinically acceptable ranges reported in previous studies for restoring femoral offset and leg length during total hip arthroplasty (THA), supporting the practical applicability of this method in preoperative planning.

**Conclusion:**

The accuracy of the present approach was comparable to that reported in healthy populations and exceeded that of previous pelvic landmark‐based regression techniques. This trochanter‐based three‐dimensional method enables clinically acceptable estimation of the FC in patients with DDH and may serve as a useful adjunct for planning of the femoral component when the native FC is difficult to identify.

## Graphical abstract

The accuracy of the trochanter‐based three‐dimensional method enables clinically acceptable estimation of the femoral head center (FC) even in patients with developmental dysplasia of the hip and may serve as a useful adjunct for planning of total hip arthroplasty when the native FC is difficult to identify.

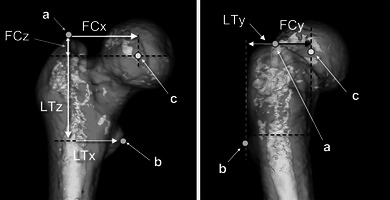

## 1 Introduction

Total hip arthroplasty (THA) involves replacing the impaired hip joint with prosthetic elements. The procedure is primarily intended to reduce pain while simultaneously improving range of motion and overall quality of life [1, 2, 3, 4, 5, 6]. In recent years, the number of THAs has increased annually, mainly in developed countries [7, 8, 9, 10, 11]. Moreover, it is expected to continue increasing in the future [12, 13]. In Japan, developmental dysplasia of the hip (DDH) remains the leading cause of THA, accounting for approximately 70%–90% of cases [14, 15, 16, 17, 18, 19]. Surgeons aim to reconstruct the original hip joint anatomy using THA; however, the hip joint after THA has a different shape from that of the original hip joint. If the opposite side is healthy, the surgeon can plan the reconstruction; however, often the opposite side cannot be the basis for reconstruction, as in bilateral cases [19, 20, 21].

In recent years, the adjustment of the global femoral offset, the sum of the femoral offset and acetabular offset, has been considered important because it can affect postoperative outcomes following THA [14, 15, 22, 23]. A suitable offset can lead to a decrease in the polyethylene liner use [24, 25], preserve the muscle power around the hip joint, such as the abductor muscles [24, 26, 27, 28], maintain the range of motion in the hip joint [28], and improve hip joint function [22, 29]. However, it is sometimes difficult to determine the original femoral head center (FC) in cases of bilateral hip osteoarthritis with severe femoral head collapse or arthritic changes. Therefore, surgeons should estimate the FC using other reference points on the femur for preoperative planning, such as in THA.

The estimation of the FC has also been carried out in earlier works, where pelvic reference landmarks—including the anterior superior iliac spine and the obturator foramen—were used in three‐dimensional models [30]. Other methods analyzed the shapes of the anterior superior iliac spine and pubic symphysis through two‐dimensional (2D) [31, 32, 33] or three‐dimensional measurements [32, 34, 35]. However, few studies have estimated the FC using reference points on the femur by the 2D [36] and 3D methods [37, 38, 39]. Several surveys have described that the direction of the tip of the lesser trochanter (LT) was strongly correlated to the direction of the femoral neck axis, and consequently with femoral neck anteversion [40, 41, 42]. Another study described that although the positional relationship between the FC and LT shows high correlation between sides, it has been reported that a significant difference exceeding 4 mm may occasionally occur [43]. In other words, the form of the proximal femur proximal to the LT is similar among individuals. Accordingly, we proposed that the location of the FC could be determined by referencing landmarks on the proximal femur, with particular emphasis on the tip of the greater trochanter (GT) and the LT as key anatomical indicators, because they can be reliably identified as appropriate reference points around the hip joint and have also been employed in previous studies [40, 41, 42]. Furthermore, the femoral center position estimated from the pelvis may differ from the true FC position from the femur due to transposition of the FC from the acetabulum, due to osteoarthritic change of the acetabulum, or the femoral head. Therefore, we considered it desirable to estimate the center position of the femoral head using reference points on the femur, which is on the same bone.

A previous study reported that the coordinate values of the FC were calculated from those of the GT and LT, with an average difference of approximately 2 mm. However, this study only included healthy volunteers [44], hindering its applicability to patients with DDH. These patients frequently exhibit nonspherical femoral heads, increased anteversion angles of the femoral neck, and reduced lengths of the femoral neck [45, 46, 47, 48]. To our knowledge, no previous study has validated a trochanter‐based three‐dimensional estimation of the FC specifically in patients with DDH.

This investigation focused on evaluating how closely the FC, estimated through three‐dimensional calculations based on the coordinate values of the greater and LT tips, corresponded to the true anatomical FC in female participants diagnosed with DDH. We hypothesized that the FC position could be calculated from the GT and LT reference points, even in DDH cases, as well as in normal cases.

## 2 Materials and Methods

### 2.1 Participants

This cross‐sectional study was conducted without any intervention. We surveyed data from 128 femurs of 84 patients with DDH, all of whom were Japanese women who underwent CPO either hemilaterally or bilaterally on their hip joint from April 1, 2010, to September 30, 2020, at our institution. In our cohort, the number of male patients during the study period was fewer than 20. This study included patients who underwent CPO at our institution, representing a surgically selected cohort. Inclusion criteria were hips diagnosed with DDH, defined as a center‐edge (CE) angle of less than 25° and Kellgren–Lawrence Grade 1 or 2. Patients with more advanced osteoarthritis (Kellgren–Lawrence grade ≥ 3) were not considered candidates for CPO and were therefore not included. Exclusion criteria included hips with a history of previous surgery, subluxation, dislocation, or joint space narrowing, as these conditions may alter femoral head morphology and compromise accurate identification of anatomical landmarks. Accordingly, the study population mainly represented mild‐to‐moderate DDH.

### 2.2 Reconstruction of the Bone Model and Measurement

**FIGURE 1:**
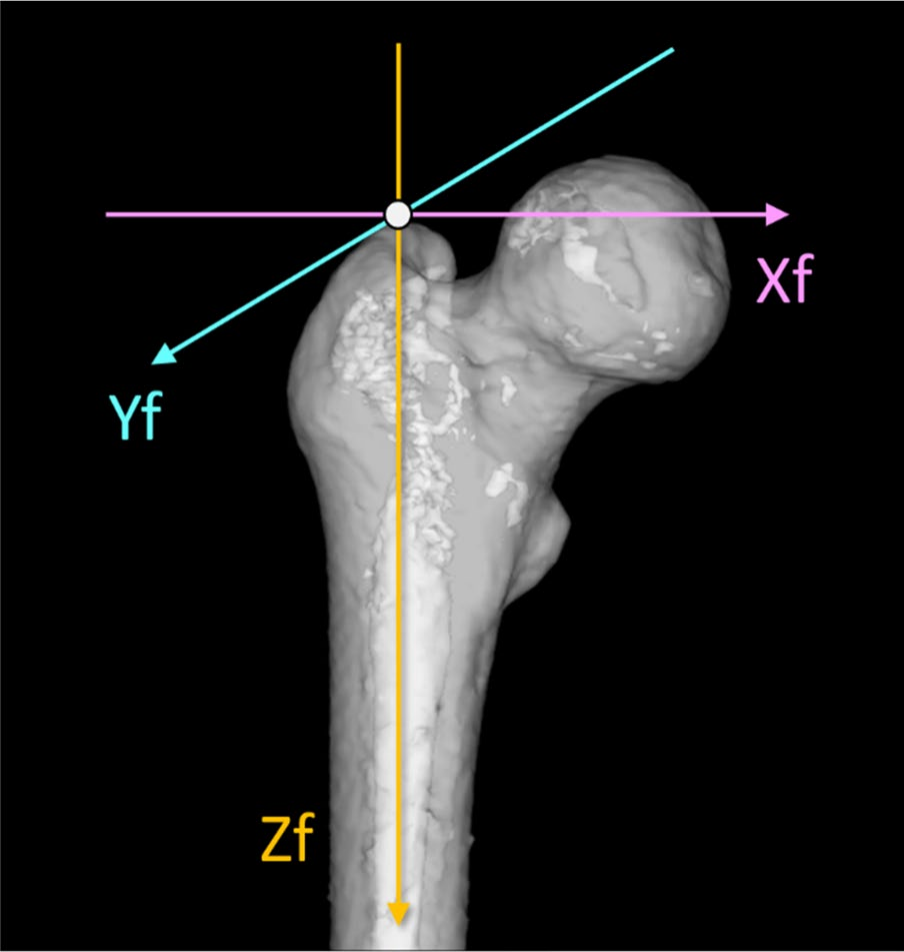
Femoral coordinate system.

Computed tomography (CT) was performed on all participants to reconstruct 3D bone models of the pelvis and femur for preoperative planning, all measurements were performed in a manner compatible with routine CT‐based preoperative planning for THA, similar to previous studies [49, 50]. A multislice CT scanner (Aquilion64; Toshiba Medical Systems, Otawara, Tochigi, Japan) was employed alongside a 64‐row detector, acquiring approximately 500 slices per participant, at a slice thickness of 1.25 mm, from the iliac crest to the fibular head. The data were accessed for research purposes between April 3 and June 1, 2023, and the authors could access to information that identify individual participants during and after data collection. Using ZedView software (version 16.0.0) [51], a 3D femoral model was constructed and aligned to the retrocondylar plane, defined by the posterior tip of the GT and the bilateral posterior condyles [52]. The craniocaudal axis was drawn from the trochanteric fossa to the distal intercondylar sulcus. Within this coordinate system, Zf was the projection of the craniocaudal axis onto the retrocondylar plane, Xf was set perpendicular to Zf in the same plane, and Yf was orthogonal to both, following established methods [53] (Figure 1).

**FIGURE 2:**
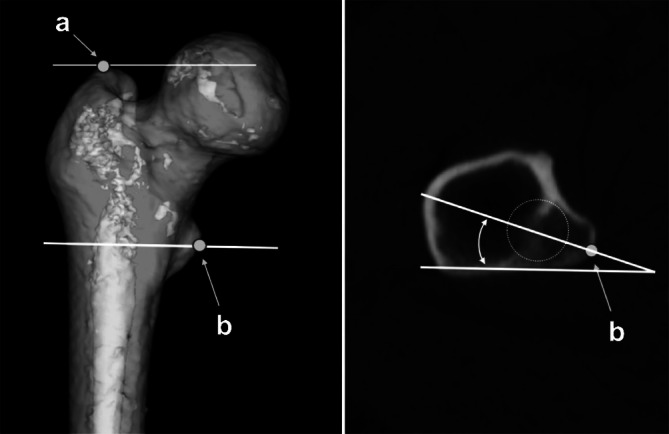
Landmarks for defining the greater and lesser trochanter (LT) tips. (a) Greater trochanter (GT) tip; (b) LT tip, following the criteria reported by Unlu et al. [42].

According to the defined coordinate axes, the tip of the GT corresponded to the most proximal location on the plane intersecting the Z‐axis at a right angle (Figure 1). In contrast, the LT tip was designated as the most prominent point on the plane orthogonal to the Zf axis (Figure 2), in line with the criteria reported by Unlu et al. [42]. The FC was defined as the geometric center of the femoral head, calculated from the three‐dimensional reconstructed model using a best‐fit sphere approach, as commonly applied in previous three‐dimensional anatomical studies.

**FIGURE 3:**
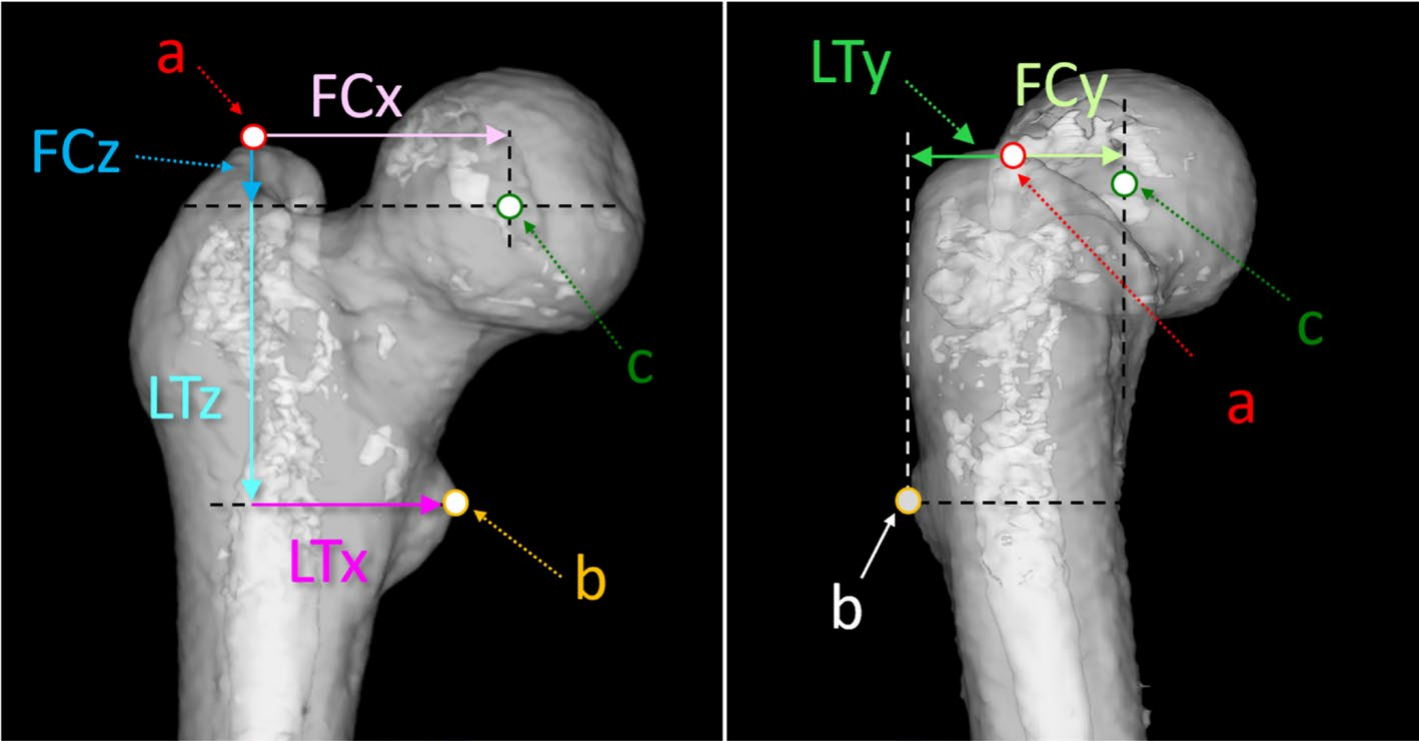
Illustration of the distance assessment between the greater and LT tips. (a) The most proximal point of the GT, defined as the measurement origin; (b) the most prominent point of the LT; (c) the calculated center of the femoral head.

For analysis, we measured the vector extending from the GT tip to the FC and described its components along each coordinate axis as FCx, FCy, and FCz (Figure 3). Likewise, the relative position of the LT tip with respect to the GT tip was represented by LTx, LTy, and LTz on the femoral Xf, Yf, and Zf axes (Figure 3). We expressed the directions in positive values for the direction from the tip of the GT, the mediolateral direction on the Xf axis, the anteroposterior direction on the Yf axis, and the craniocaudal direction on the Zf axis. To estimate the FC point, we calculated the coordinate values of the FC point in the Xf, Yf, and Zf directions of FC, FCx, FCy, and FCz, respectively, relative to the tip of the GT, using the coordinate values of the tip of the LT, LTx, LTy, and LTz with multiple regression analysis. The estimation errors of FCx, FCy, and FCz were examined using the *t*‐test for differences, the *F*‐test for dispersion, and the Shapiro–Wilk test for normality of distribution. All analyzes were conducted on a per‐femur basis.

### 2.3 Statistical Analysis

To assess the reproducibility of the measurements, intra‐ and inter‐rater reliabilities were examined through intraclass correlation coefficients (ICCs) in a two‐way, mixed‐effects model. For intra‐rater testing, each parameter was measured twice by the same examiner with a minimum interval of 1 week between sessions. Inter‐rater reliability was verified by having two independent investigators perform repeated assessments. Statistical procedures were conducted with SPSS software, version 26 (SPSS Inc., Chicago, IL, USA). A significance threshold of *p* < 0.05 was applied for regression modeling and ICC calculations.

### 2.4 Ethics

The Institutional Review Board of Niigata University approved the study design (approval no. 2025‐0250) and waived the requirement for informed consent owing to the cross‐sectional, retrospective, and non‐interventional nature of the study. This investigation followed established ethical standards and regulatory guidelines.

## 3 Results

### 3.1 Demographic Characteristics

The demographic data of the participants are presented in Table 1.

**TABLE 1:** Details of the participants.

Age (years)	36.9 ± 8.0
Body height (cm)	158.2 ± 6.4
Body weight (kg)	52.1 ± 7.0
Body mass index (kg/m^2^)	20.8 ± 2.8

*Note:* All values are expressed as mean ± standard deviation.

### 3.2 Regression Formulas for FC Estimation

FCz had a negative value, indicating that the FC was positioned below GT in the coronal and sagittal planes of the femoral coordinate system (Table 2).

**TABLE 2:** Comparative values for the positional relationship of the greater trochanter tip with respect to the femoral head center (FC) and the lesser trochanter.

FCx (mm)	32.7 ± 6.5
FCy (mm)	25.3 ± 6.9
FCz (mm)	3.2 ± 4.6
LTx (mm)	34.2 ± 4.7
LTy (mm)	4.3 ± 7.3
LTz (mm)	45.7 ± 9.2

*Note:* All values are expressed as mean ± standard deviation.

The formulae calculated from the multivariate regression were as follows: FCx = 0.568 × LTX‐0.566 × LTY+15.760, correlation coefficient was 0.788 (*p* < 0.01), FCy = 0.770 × LTY+22.072, correlation coefficient was 0.727 (*p* < 0.01), FCz = −0.462 × LTZ+24.302, correlation coefficient was 0.875 (*p* < 0.01) (Table 3).

**TABLE 3:** Formulas were calculated with the multiple regression analysis.

Formulae	Adjusted *R* ^2^
FCx = 0.568 × LT_X_ − 0.566 × LT_Y_+15.760	0.617
FCy = 0.770 × LT_Y_ + 22.072	0.629
FCz = −0.462 × LT_Z_ + 24.302	0.766

### 3.3 Estimation Accuracy

The error between the actual and calculated values was 2.81 mm for FCx, 3.65 mm for FCy, and 2.02 mm for FCz (Table 4). Moreover, the root mean square error (RMSE) was 3.42 mm for FCx, 4.41 mm for FCy, and 2.57 mm for FCz, indicating that the estimation error remained within 5 mm in all directions (Table 4).

**TABLE 4:** Differences between the measured and calculated values.

	Difference	Root mean square error
FCx (mm)	2.81 ± 1.95	3.42
FCy (mm)	3.65 ± 2.47	4.41
FCz (mm)	2.02 ± 1.59	2.57

*Note:* All values are expressed as mean ± standard deviation.

**FIGURE 4:**
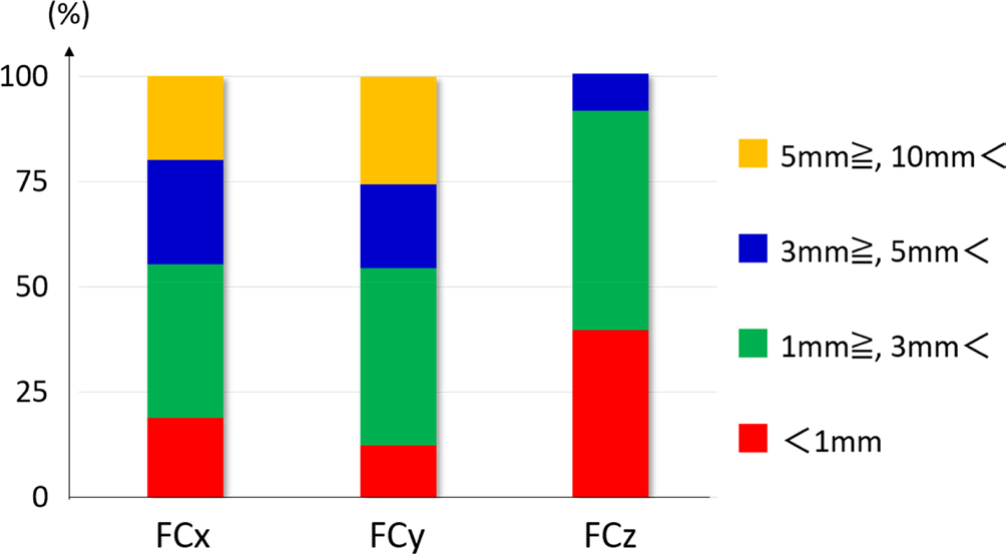
Plot showing the differences between actual measurements and estimated coordinates. The deviations in FCx and FCz did not exceed 5 mm, indicating high accuracy of estimation (*n* = 128).

The formulas could estimate 54.7%–91.7% of each length within 3 mm and 75.0%–100% of each length within 5 mm (Figure 4).

### 3.4 Reliability and Statistical Validation

We also assessed multicollinearity among LTx, LTy, and LTz. The variance inflation factor values were 1.006 for LTx and LTy in the FCx model, 1.000 for LTy in the FCy model, and 1.000 for LTz in the FCz model. Concerning the Shapiro–Wilk test, the *p* value was 0.228 for FCx, 0.322 for FCy, and 0.188 for FCz, indicating no significant deviation from normality. For all measurements, we obtained ICCs > 0.8 for all measurements, FCx, FCy, FCz, LTx, LTy, and LTz, indicating high intra‐ and interobserver reliabilities.

## 4 Discussion

### 4.1 Accuracy and Clinical Relevance of the Estimation Method

We observed that the mean error between observed values and calculated estimates was limited to roughly 2–3 mm. This degree of accuracy supports the use of a regression equation for estimating the FC coordinates with practical reliability. Previously, several studies estimated FC using 2D procedures with plane radiographs [31, 32, 33, 53, 54, 55, 56, 57]. Even with 2D methods, researchers have found that they can estimate the hip joint center accurately within 5 mm. The estimates can vary between 10 and 20 mm in the side‐to‐side and up‐and‐down directions. However, examining the anteroposterior dimension using 2D analysis was impossible. These 2D methods were considered easy to perform and practical; however, it was difficult to assess changes such as the decrease in FCx observed in this study, which may be affected by stem anteversion. If the stem anteversion increases, FCx decreases, preventing the surgeons from restoring FC. Moreover, it is difficult to determine femoral reference points, such as the tips of the GT and LT. Furthermore, 2D methods may be greatly affected by the position of the femur and pelvis, such as internal or external rotation [31, 56]. Therefore, a 3D estimation method is considered valuable. During THA planning, when the hip center is translated medially due to the positioning of the acetabular component, surgeons can adjust the global and femoral offsets by considering the estimated value of FCx [14, 15, 23].

### 4.2 Comparison With Previous Studies

Several investigations have sought to determine the position of the FC by referencing bony landmarks of the pelvis, such as the anterior superior iliac spine and the margins of the obturator foramen [30, 34, 35]. When these pelvic reference points were employed, the calculated values typically deviated from the true coordinates by 3–5 mm.

However, the estimation of the FC using the GT and LT reference points was similar to that reported in healthy participants using the 3D method [44]. Therefore, this method applies not only to healthy participants but also to patients with DDH. However, the estimation errors seemed to be slightly larger than those in the healthy participants, suggesting the possibility of variations in bone geometry compared to the healthy participants.

### 4.3 Clinical Implications for THA Planning

In this study, for the estimation of the femoral head center, the correlation in FCy, representing the anteroposterior direction of the femur, was relatively low, similar to that in healthy participants. Several reports have suggested that restoration of global femoral offset (X‐direction in this study) contributes to improved postoperative hip function through preservation of abductor muscle strength [5, 6]. Leg lengthening in the cephalocaudal direction (Z‐direction in the current study) can influence the postoperative outcome of THA [39], whereas the anteroposterior offset (Y‐direction in the current study) has less influence on the postoperative outcome [14]. Conversely, it was also stated that restoring the original anteversion angle as much as possible, even in DDH cases, is critical for optimizing functional recovery and implant stability [53]. The restoration of the anteversion with the femoral component, which can be associated with the Y‐direction, is considered to affect the dislocation following implant impingement after THA and wear of the liner [58, 59, 60]. Therefore, we suggest that during preoperative planning of THA, it would be necessary to adjust to the estimated FCx and FCz first, and then align in the Y‐direction, rather than attempting to align all three forcibly from the beginning. This is because FCx is closely related to global femoral offset and FCz to leg length discrepancy, both of which require an accuracy of approximately 5 mm [14, 19, 22, 23, 24]; previous studies have suggested that restoration of femoral offset and leg length within approximately 5 mm is associated with favorable outcomes after THA [14, 19, 22, 23, 24, 25]. In this context, the estimation error observed in the present study may be considered relatively small. However, the direct clinical impact of this level of error remains unclear and requires further investigation. In contrast, the estimation error for FCy, which is associated with femoral anteversion, was approximately 3.6°, indicating relatively greater variability in the anteroposterior direction. From the perspective of anterior–posterior offset on the femoral side, offset magnitude does not directly influence clinical outcomes on its own [19]. Although FCy undoubtedly contributes to femoral anteversion, its impact varies depending on the native anteversion angle, neck length, and offset. Therefore, it is difficult to quantify the isolated effect of FCy, and it remains uncertain whether this degree of error is clinically acceptable.

### 4.4 Strengths, Limitations, and Future Perspectives

To our knowledge, there were few reports of FC estimation by a 3D method using the reference points of the femur in patients with DDH. Moreover, our method is considered more accurate than those calculated using the reference points on the pelvis, representing a major strength of this study. From a surgical perspective, prioritizing accurate restoration of FCx and FCz may be more critical than FCy, given their direct influence on global femoral offset and leg length.

This research is not without limitations. One important concern is that the cohort comprised only 84 individuals, providing data from a total of 128 femurs in a single institution, which may restrict the generalizability of the findings. Moreover, acetabular dysplasia occurs significantly more often in women, with reported female‐to‐male ratios ranging from approximately 2:3 up to 9:1 [61, 62, 63, 64]. In our own cohort, the number of male patients during the study period was fewer than 20. Consequently, this analysis had to be restricted to female patients, which represents one of the limitations of this study. Because this study was based on a retrospective surgical cohort, the number of patients excluded at each stage could not be fully determined, which may limit the transparency of the selection process. Because the present study included only mild‐to‐moderate DDH cases, the influence of disease severity on estimation accuracy could not be evaluated. Future studies including a broader spectrum of DDH severity, such as high‐grade cases based on the Crowe classification, are warranted to evaluate the robustness of this method. In addition, no independent validation cohort was used in this study, and therefore the generalizability of the regression models remains uncertain. The possibility of overfitting cannot be excluded, and external validation using independent datasets is warranted. However, in DDH cases, the femoral head may not be perfectly spherical, which could introduce a small degree of error in defining the reference center based on a best‐fit sphere approach. These factors should be considered when interpreting the present findings. Second, there were limitations inherent to the study design and the risk of bias. Third, all the participants were Japanese females, limiting the generalizability of the findings to other populations. Fourth, we excluded cases with previous surgery, subluxation, dislocation, and joint space narrowing. Postoperative conditions may alter the original position of the FC, and the degree of subluxation can also affect femoral morphology [65]. Moreover, deformities may occasionally hinder the accurate identification of the femoral center, potentially introducing bias into our findings. Consequently, we believe it is prudent to exclude such cases from our analysis. Furthermore, our method of utilizing the tips of the greater and LTs may present complexities. Although the results align closely with those obtained from healthy participants across three dimensions, indicating its relevance for healthy individuals and patients with DDH, the ease of implementation remains uncertain. In recent years, software using artificial intelligence technology has been developed to automatically calculate the center position of the femoral head and diagnose DDH from pain radiographs [66, 67] in 2D radiographs. However, these software tools can currently only calculate the FC position based on the contour of the femoral head, not the original FC position of a crushed or flattened femoral head. In the future, it may be possible to estimate these positions more accurately, as the tips of the GT and LT could be determined not only by plain radiographs, because 3D CT reconstruction may not be accessible in every institution in daily clinical practice, but also by 3D images through the development of artificial intelligence techniques. This investigation was confined to estimating the FC based on proximal femoral morphology, and its potential implications for improved surgical outcomes have not yet been substantiated. A central goal of the investigation is to propose estimation procedures that are efficient and uncomplicated, making them suitable for everyday use in clinical practice.

## 5 Conclusions

This trochanter‐based three‐dimensional method may provide a practical approach for estimating the FC to assist femoral component positioning in Japanese female patients with mild‐to‐moderate DDH; however, further validation is required.

## Author Contributions

**Atsushi Sakagami:** conceptualization, methodology, resources, data curation. **Norio Imai:** writing – original draft, writing – review and editing, methodology, conceptualization, investigation, formal analysis, resources. **Daisuke Homma:** conceptualization, validation, methodology. **Yuki Hirano:** conceptualization, methodology, validation. **Yoji Horigome:** conceptualization, writing – review and editing. **Yuki Komuta:** conceptualization, methodology, validation. **Hiroyuki Kawashima:** supervision, writing – review and editing.

## Funding

This research received no specific grant from any funding agency in the public, commercial, or not‐for‐profit sectors.

## Ethics Statement

This study was approved by the Institutional Review Board of Niigata University (approval number: 2025–0250). The requirement for informed consent was waived owing to the retrospective, cross‐sectional, and non‐interventional nature of the study.

## Consent

The authors have nothing to report.

## Conflicts of Interest

The authors declare no conflicts of interest.

## Data Availability

The data that support the findings of this study are available from the corresponding author upon reasonable request.
